# MicroRNA-27a promotes proliferation and suppresses apoptosis by targeting *PLK2* in laryngeal carcinoma

**DOI:** 10.1186/1471-2407-14-678

**Published:** 2014-09-18

**Authors:** Yuan Tian, Shuang Fu, Guang-Bin Qiu, Zhen-Ming Xu, Ning Liu, Xiao-Wen Zhang, Sheng Chen, Ye Wang, Kai-Lai Sun, Wei-Neng Fu

**Affiliations:** Department of Medical Genetics, China Medical University, Shenyang, 110001 People’s Republic of China; Department of Hematology Laboratory, Shengjing Hospital of China Medical University, Shenyang, 110011 People’s Republic of China; Department of Laboratory Medicine, No. 202 Hospital of PLA, Shenyang, 110003 People’s Republic of China; Department of Otolaryngology, No. 463 Hospital of PLA, Shenyang, 110007 People’s Republic of China

**Keywords:** Laryngeal squamous cell carcinoma, miR-27a, *PLK2*, Apoptosis, Proliferation

## Abstract

**Background:**

miRNA-27a has been confirmed as an important regulator in carcinogenesis and other pathological processes. Whether and how it plays a role in the laryngeal carcinoma is unknown.

**Methods:**

Mature miRNA-27a expression in laryngeal cancer was detected by qRT-PCR. Gain-of-function studies using mature miR-27a were performed to investigate cell proliferation and apoptosis in the Hep2 cells. In silico database analysis and luciferase reporter assay were applied to predict and validate the direct target, respectively. Loss-of-function assays were performed to investigate the functional significance of the miR-27a target gene. qRT-PCR and Western blot were used to evaluate mRNA and protein levels of the target, respectively.

**Results:**

miR-27a was significantly up-regulated in the laryngeal tumor tissues compared to the adjacent non-tumor tissues. In silico database analysis result revealed that *PLK2* is a potential target of miR-27a. luciferase reporter assay result showed the direct inhibition of miR-27a on PLK2-3′UTR. In the cases with miR-27a up-regulation, *PLK*2 protein expression level was significantly lower in cancer tissues than that in the adjacent non-tumor tissues, which showed a negative correlation with miR-27a expression level. Both miR-27a and knockdown of *PLK*2 caused the increase of the cell viability and colony formation and inhibition of the late apoptosis in the Hep2 cell lines. Moreover, miR-27a but not PLK2 also repressed the early apoptosis in the Hep2 cells. Additionally, no alteration of the Hep2 cell cycle induced by miR-27a was detected.

**Conclusions:**

miR-27a acts as an oncogene in laryngeal squamous cell carcinoma through down-regulation of *PLK2* and may provide a novel clue into the potential mechanism of LSCC oncogenesis or serve as a useful biomarker in diagnosis and therapy in laryngeal cancer.

**Electronic supplementary material:**

The online version of this article (doi:10.1186/1471-2407-14-678) contains supplementary material, which is available to authorized users.

## Background

Laryngeal squamous cell carcinoma (LSCC) is one of the most common head and neck cancers in the world. In China, the incidence of LSCC has been rising gradually, especially in the northeast part. The potentially high incidence of morbidity and incommensurably low cure rate, as expected, require searching for new diagnostic procedures and carcinogenic factors of LSCC [1–3]. Although great progress has been achieved in the study on laryngeal cancer, there are no ideal biomarkers for the determination of prognosis and the guidance of treatment in laryngeal cancer patients. Presently, much work is focused on the identification of useful biologic and molecular markers in the diagnosis and therapy of LSCC [4, 5].

MicroRNAs (miRNAs), a 20–23 nt functional RNA molecule, are a class of short non-coding RNAs and play important regulatory roles by sequence-specific base pairing on the 3′ untranslated region (3′-UTR) of target messenger RNAs (mRNAs), in promoting mRNA degradation or inhibiting translation [6]. Increasing evidence showed that miRNAs have significant roles in diverse biological processes [7]. microRNAs have been functionally classified as proto-oncogenes or tumor suppressors and are aberrantly expressed in different cancers including leukemia [8, 9], lymphoma [10], breast cancer [11, 12], colorectal cancer [13], lung cancer [14, 15], liver cancer [16, 17], and head and neck cancer [18–21] ^.^ It has been suggested that miRNA may be a molecular target for cancer diagnosis and therapy.

As one of hundreds of microRNAs, miRNA-27a (miR-27a) has been confirmed as an important regulator in carninogenesis and other pathological processes. The oncogenic role of miR-27a has been verified by several studies. For examples, miR-27a was significantly up-regulated in renal cell carcinoma [22], cervical cancer [23], gastric adenocarcinoma [24] and breast cancer [25, 26]. miR-27a was reported to be involved in other diverse processes, such as osteoarthritis pathological process [27], viral infections [28], adipocyte differentiation [29], fat metabolism and cell proliferation [30], and multidrug resistance [31]. However, the relationship of miR-27a to LSCC and its role in the genesis of LSCC are not yet described. In this study, we demonstrated that miR-27a, a frequently up-regulated miRNA in LSCC, could induce cell proliferation and repress apoptosis in the Hep2 cells. Moreover, *PLK2* was characterized as a direct target of miR-27a.

## Methods

### Patient tissues and cell lines

Tissue specimens (tumor tissue and paired adjacent tissue) from 67 LSCC patients were used in the study. All of the patients provided written informed consent, and approval for the study was received from the Ethics Committee of China Medical University. Verification of the specimens was performed by a pathologist and the samples were immediately frozen at -80°C after been removed from the patients. The human Hep2 (laryngeal cancer) and HEK293 (embryonic kidney) cell lines were obtained from the Cell Biology Institute of Shanghai, Chinese Academy of Science and were maintained in RPMI 1640 (GIBCO, Los Angeles, CA) with 10% fetal bovine serum (Hyclone, Logan, USA), 100 units/ml penicillin and 100 μg/ml streptomycin in a humidified atmosphere at 37°C in 5% CO_2_.

### Gene transfection

Cell-based experiments were carried out by transfection of 20nM miRNA duplex (GenePharma, Shanghai, China), non-relative control RNA duplex (NC duplex, GenePharma) and small interfering RNA (siRNA, GenePharma) into the Hep2 cells using Lipofectamine™ 2000 in accordance with the manufacturer’s procedure. The sequences of the corresponding small non-coding RNAs are as follows: miR-27a mimics: 5′-UUCACAGUGGCUAAGUUCCGC-3′; miR-27a inhibitor: 5′-GCGGAACUUAGCCACUGUGAA-3′, mimics NC: 5′-UUCUCCGAACGUGUCACGUTT-3′, inhibitor NC: 5′-CAGUACUUUUGUGUAGUACAA-3′, NC: 5′-GGCUACGUCCAGGAGCGCA CC-3′and siPLK2: 5′-CACAGAAGGAGAACGAUAUTT -3′.

### Fluorescence detection

Cells were transfected by the FAM-labeled miR-27. After cultured for 6 h, the cells were visualized by fluorescence microscope (BX51TF, OLYMPUS, Japan) to evaluate the transfection efficency.

### Transcriptional expression assay

Total RNA was extracted from the specimens and the cells using Trizol (Takara, Dalian, China) according to the manufacturer’s instructions. MicroRNA was separated using a miRcute miRNA isolation kit (Tiangen, Bejing, China). The concentrations of small and total RNA were measured by reading the absorbance at OD260/280 nm.

To test the expression of miR-27a and *PLK2* mRNA in the LSCC tissues and the cell lines, qRT-PCR was carried out using the ABI 7500 Real Time PCR system (Applied Biosystems, Foster City, CA, USA). For the mature miR-27a detection, reverse transcription and quantitative PCR were performed using the One Step PrimeScript miRNA cDNA Synthesis Kit (Takara, Dalian, China) and SYBR® Premix Ex Taq™ II (Takara, Dalian, China). U6 small nuclear RNA (snRNA) expression was assayed for normalization. A miR-27a specific primer and a universal reverse primer RTQ-UNIr were used for the amplification. Primer sequences for miR-27a and RTQ-UNIr are 5′-TTCACAGTGGCTAAGTTCCGC-3′ and 5′-CGAATTCTAGAGCTCGAGGCAGGCGA CATGGCTGGCTAGTTAAGCTTGGTACCGAGCTCGGATCCACTAGTCC (T)-3′, respectively. Primer sequences for U6 are as follows: F-5′-CTCGCTTCGGCAGCACA-3′, R-5′-AACGCTTCACGAATTTGCGT-3′. The PCR conditions for miR-27a and U6 snRNA are 95°C for 30 sec, followed by 40 cycles of 95°C for 5 sec and 60°C for 34 sec. To detect *PLK2* mRNA, SYBR® Premix Ex Taq™ II (Takara, Dalian, China) was used. Primers for *PLK2* are as follows: F-5′-TCAGCAACCCAGCAAACACAGG-3′ and R-5′-TTTCCAGACATCCCCGAAGAACC-3′. Primers for *GAPDH* are as follows: F-5′-TTGCTAGAGACCGAGTGTCC-3′ and R-5′-CTTTGTGGCTTTCTTCATGG-3′. The PCR conditions for the *PLK2* and *GAPDH* are 95°C for 30 min, 40 cycles of 95°C for 5 sec and 60°C for 34 sec. ΔCt was calculated by subtracting the Ct of U6 or GAPDH mRNA from the Ct of the RNAs of the interest. ΔΔCt was then calculated by subtracting the ΔCt of the negative control from the ΔCt of the samples. The fold change in microRNA or mRNA was calculated according to the equation 2^-ΔΔCt^.

### In vitro cell proliferation and colony formation assays

For cell proliferation analysis, 2-3 × 10^3^ of the Hep2 cells after transfection were plated into 96-well plates. Cells were then cultured for 1, 2, 3, 4 and 5 days, respectively. The absorbance at 570 nm was measured after incubation of the cells with 100 μl sterile MTT dye (0.5 mg/ml, Sigma) for 4 h at 37°C and 150 μl DMSO for 15 min. Then the cell growth curve was constructed by using OD570 nm as ordinate axis.

In the colony formation assay, 3-5 × 10^3^ of the Hep2 cells at twelve hours after transfection were seeded in a 60-mm Petri dish in triplicate and maintained in RPMI 1640 (GIBCO, Los Angeles, CA) with 10% fetal bovine serum. After 14 days, the colonies were fixed with methanol for 30 min, stained with hematoxylin for 20 min, and scored using a microscope. Colony formation for each condition was calculated in relation to the values obtained for mock and the scramble-treated control cells.

### Flow cytometry-based apoptosis and cell cycle analysis

Cells were grown in 6-well plates to about 60% confluence and transiently transfected with the desired miRNA and siRNA-PLK2 reagents. The cells were digested and collected after 48 h or 72 h post-transfection, and washed with PBS twice. For apoptosis detection, the cells were treated by Annexin V-EGFP Apoptosis Detection Kit according to the manufacturer’s instructions (KeyGEN, Nanjing, China). For cell cycle analysis, the cells were resuspended in PBS and then fixed in ethanol at -20°C for at least 12 hours. The cells were washed with PBS and resuspended again in staining solution (50 μg/mL of propidium iodide, 1 mg/mL of RNase A in PBS). After the treatment, the cells (1 × 10^5^) were then analyzed with a flow cytometer (FACS calibur, Becton-Dickinson, USA).

### pGL3-PLK2-3′-UTR luciferase reporter assay

The prediction of *PLK2* mRNA as a target of miR-27a was made with miRanda (http://cbio.mskcc.org/cgi-bin/mirnaviewer/mirnaviewer.pl?type=miRanda), Pictar (http://pictar.mdc-berlin.de/) and TargetScan (http://www.targetscan.org/) programs. pGL3-PLK2-3′UTR and pGL3-PLK2-3′UTR-mut plasmids were obtained from GENECHEM (Shanghai, China). The HEK293 cells seeded in 96-well plate in triplicate were cotransfected with pGL3-PLK2-3′UTR or pGL3-PLK2-3′UTR-mut and miRNA-27a mimic or non-relative control RNA duplex (NC duplex, GenePharma) by using Lipofectamine™ 2000 in accordance with the manufacturer′s procedure. pRL-TK (Promega Corporation) was transfected as a normalization control. The cells were collected at 24 h after transfection and luciferase activity was measured using a dual-luciferase reporter assay kit (Promega Corporation) and recorded by Chemiluminescence meter (Promega Corporation).

### Western blotting

Protein was extracted from the cells at 48 h after transfection and LSCC tissues using a protein extraction reagent (Beyotime, Shanghai, China) and protein concentration was measured using the BCA Protein Assay kit (Beyotime). 50 μg of the extracts were separated on 10% SDS-PAGE and transferred to PVDF membrane. The membrane was then blocked with 5% non-fat milk and incubated with anti-PLK2 antibody (1:500 dilution; Abcam, USA) followed by horseradish peroxidase-conjugated antibody (1:2000 dilution; ZhongShan, China). Detection was performed by enhanced chemi-luminescence (ECL) using a Western blotting immunological reagent (Santa Cruz Biotechnology) according to the manufacturer’s instructions. β-actin was used as a reference protein and determined following the same procedure as above.

### Statistical analysis

Unless otherwise stated, each experiment was performed for a minimum of three times. Data were subjected to statistical analysis by SPSS 16.0 software and shown as mean ± standard error of the mean (SEM). A paired-samples *T*-test was used to analyze differences in miR-27a expression between LSCC tissues and paired adjacent tissues. Pearson’s product–moment correlation coefficient was used to assess the correlation between PLK2 protein and miR-27a levels in LSCC. The results of cell-based experiments were analyzed by an independent samples *T*-test and one-way ANOVA. P < 0.05 was considered statistically significant.

## Results

### miR-27a is up-regulated in the human LSCC specimens and Hep2 cells

**Figure 1:**
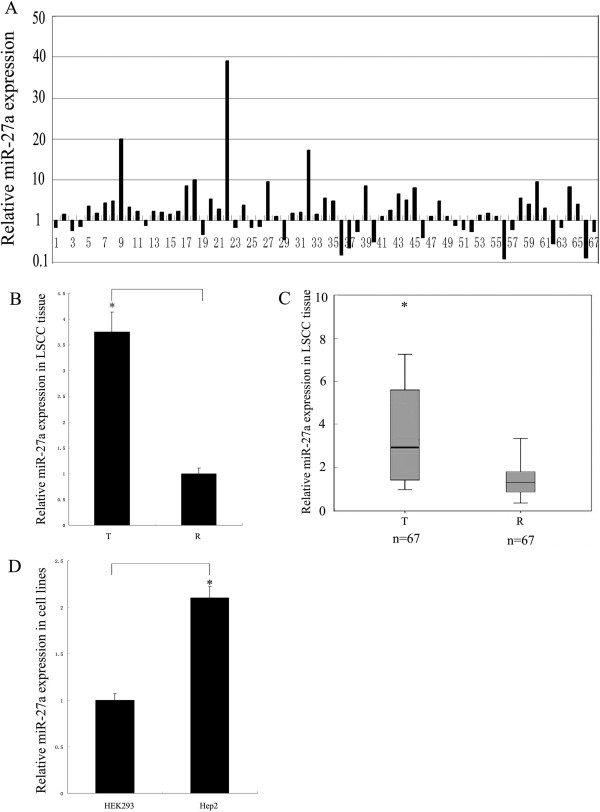
**miR-27a expression in LSCC tissues and Hep2 cells by qRT-PCR. (A)** Relative expression of miR-27a in LSCC tissues. Y-axis indicates the ratio of relative miR-27a expression in cancer tissue to that in the paired adjacent tissue. The relative expression was calculated as the ratio of miR-27a to the internal control using the equation RQ = 2^–ΔΔCT^ in each sample. The digit on X-axis show the number of the paired samples used in the study. **(B)** Statistical analysis of miR-27a expression in LSCC. T and R indicate cancer tissue and the paired adjacent tissue, respectively. **(C)** Normalized box plot for miR-27a expression in LSCC. T and R indicate cancer tissue and the paired adjacent tissue, respectively. **(D)** miR-27a expression in the cell lines. Y-axis indicates relative miR-27a expression which was calculated as the ratio of miR-27a to the internal control using the equation RQ = 2^–ΔΔCT^. Data were expressed as the mean ± SD from three independent experiments. P < 0.05 is indicated as symbol*.

We explored the expression of miR-27a in LSCC by qRT-PCR in 67 paired of fresh LSCC tissues and the paired adjacent tissues. Amplification plot and dissociation curve of miR-27a showed that the qRT-PCR conditions were reliable (see Additional file 1: Figure S1A and B). As shown in Figure 1A, 65.7% (44/67) cases of cancer tissues showed high concentrations of miR-27a with respect to the paired controls. General expression level of miR-27a was significantly up-regulated in LSCC tissues compared to the paired counterparts (*T*-test, p < 0.05) (Figure 1B and C). Meanwhile, the expression level of miR-27a in the Hep2 cells was significantly increased compared to that in the HEK293 cells (Figure 1D). Together, these results suggest that miR-27a plays an important role in LSCC.

### miR-27a promotes proliferation and suppresses apoptosis in the Hep2 cells

**Figure 2:**
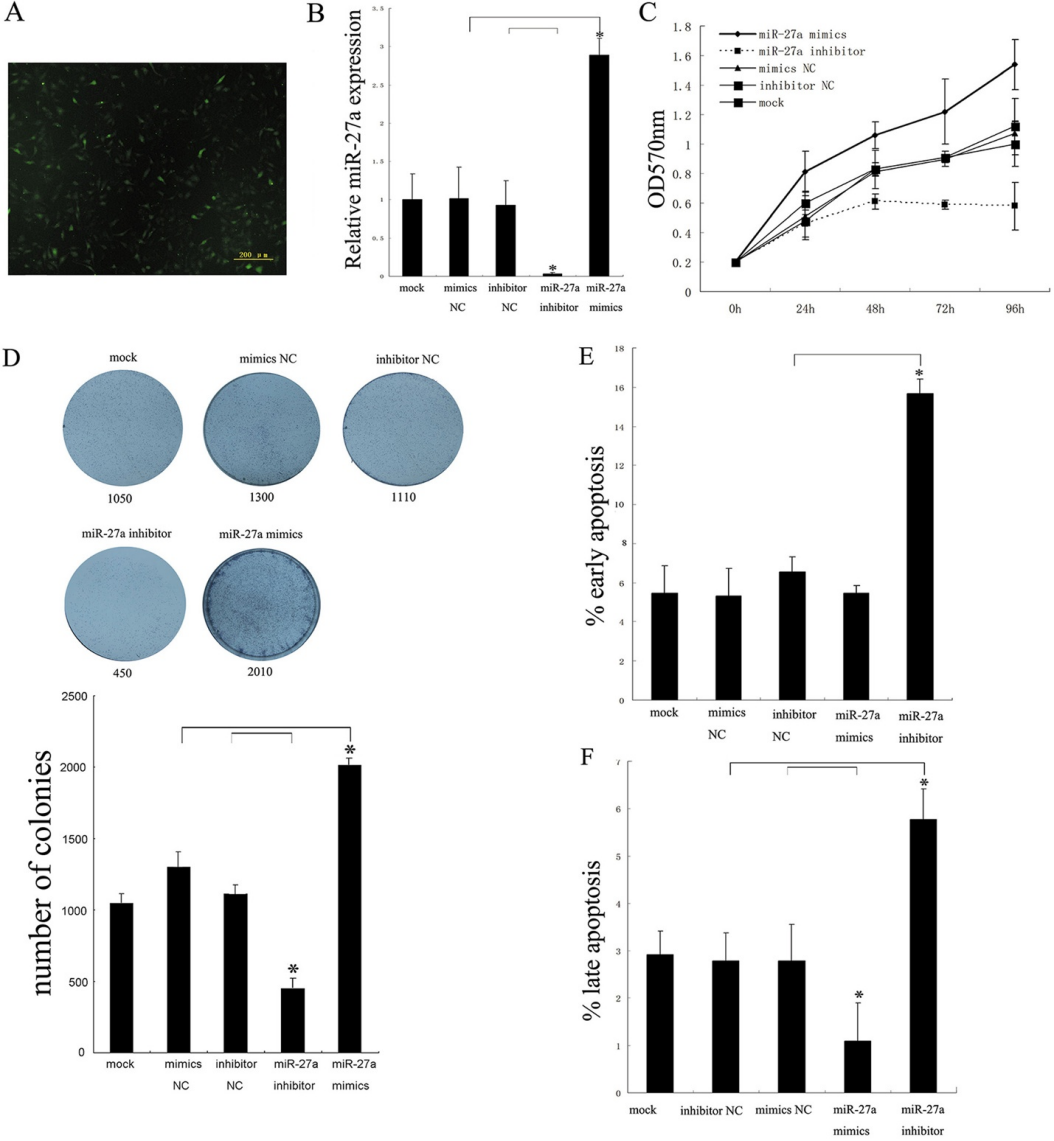
**Regulation of miR-27a in the proliferation and apoptosis in the Hep2 cells. (A)** Transfection efficiency of miR-27a in the Hep2 cells observed by fluorescence microscope. miR-27 labeled with FAM was transfected into the Hep2 cells and the fluorescence protein at 6 hr after transfection was detected using a fluorescence microscope. **(B)** Transfection efficiency of the corresponding miRNAs in the Hep2 cells by qRT-PCR. After transfection, the expression of miR-27a or the control miRNAs in the Hep2 cells were monitored using qRT-PCR. **(C)** Effect of miR-27a on the Hep2 cell proliferation measured by the MTT assay. Hep2 cells were transfected with miR-27a or the control miRNAs in the Hep2 cells and the cell proliferation was detected using the MTT assay. **(D)** Effect of miR-27a on the Hep2 cell proliferation measured by the colony formation assay. Hep2 cells were transfected with miR-27a or the control miRNAs in the Hep2 cells and the cell proliferation was detected using the colony formation assay. **(E)** Effect of miR-27a on the early apoptosis of the Hep2 cell lines. Hep2 cells were transfected with miR-27a or the control miRNAs and treated by Annexin V-EGFP apoptosis detection kit. The early apoptotic percentages of the Hep2 cells in different groups were monitored by flow cytometry. **(F)** Effect of miR-27a on the late apoptosis of the Hep2 cell lines. Hep2 cells were transfected with miR-27a or the control miRNAs and treated by Annexin V-EGFP apoptosis detection kit. The late apoptotic percentages of the Hep2 cells in different groups were monitored by flow cytometry. Data were expressed as the mean ± SD from three independent experiments. P < 0.05 is indicated as symbol*.

The Immunofluorescence result displayed that lots of cells were stained by fluorescent material (Figure 2A). As shown in Figure 2B, miR-27a mimic was significantly expressed in the Hep2 cells and its inhibitor significantly reduced miR-27a expression levels, suggesting that miR-27a mimics and miR-27a inhibitor have been successfully introduced into the cells and the following detection is credible. To assess the effect of miR-27a on cell growth, the MTT and colony formation assay were performed in the Hep2 cells after the corresponding transfection experiments. MTT assay results indicated that the cells transfected with miR-27a mimic showed significantly higher proliferation ability than those in the control groups, however the cells transfected with miR-27a inhibitor showed opposite effect on proliferation ability compared to those in the control groups, especially on day 4 and 5 after transfection (Figure 2C). The colony formation assay results displayed that the Hep2 cells transfected with miR-27a mimic showed much more and larger colonies compared to the control groups, but the cells transfected with miR-27a inhibitor revealed much fewer and smaller colonies compared to the controls (Figure 2D).

Flow cytometry assay results indicated that the significant increase in the early apoptosis was observed in the Hep2 cells transfected with the miR-27a inhibitor, whereas no significant alteration in early apoptosis was detected in the Hep2 cells transfected with miR-27a mimics (Figure 2E), the reason for which might be the abundant expression of internal miR-27a in the Hep2 cells (Figure 1D). As for the effect of miR-27a on the late apoptosis, the results displayed that miR-27a also significantly suppressed the late apoptosis of the Hep2 cells and its inhibitor rescued the effect significantly (Figure 2F). Meanwhile, no significant difference in the cell cycle of the Hep2 cells was observed either transfected with miR-27a mimic or its inhibitor (see Additional file 2: Figure S2), which indicates that miR-27a does not affect the cell cycle checkpoints of the Hep2 cells.

### *PLK2*mRNA is a direct target of miR-27a

**Figure 3:**
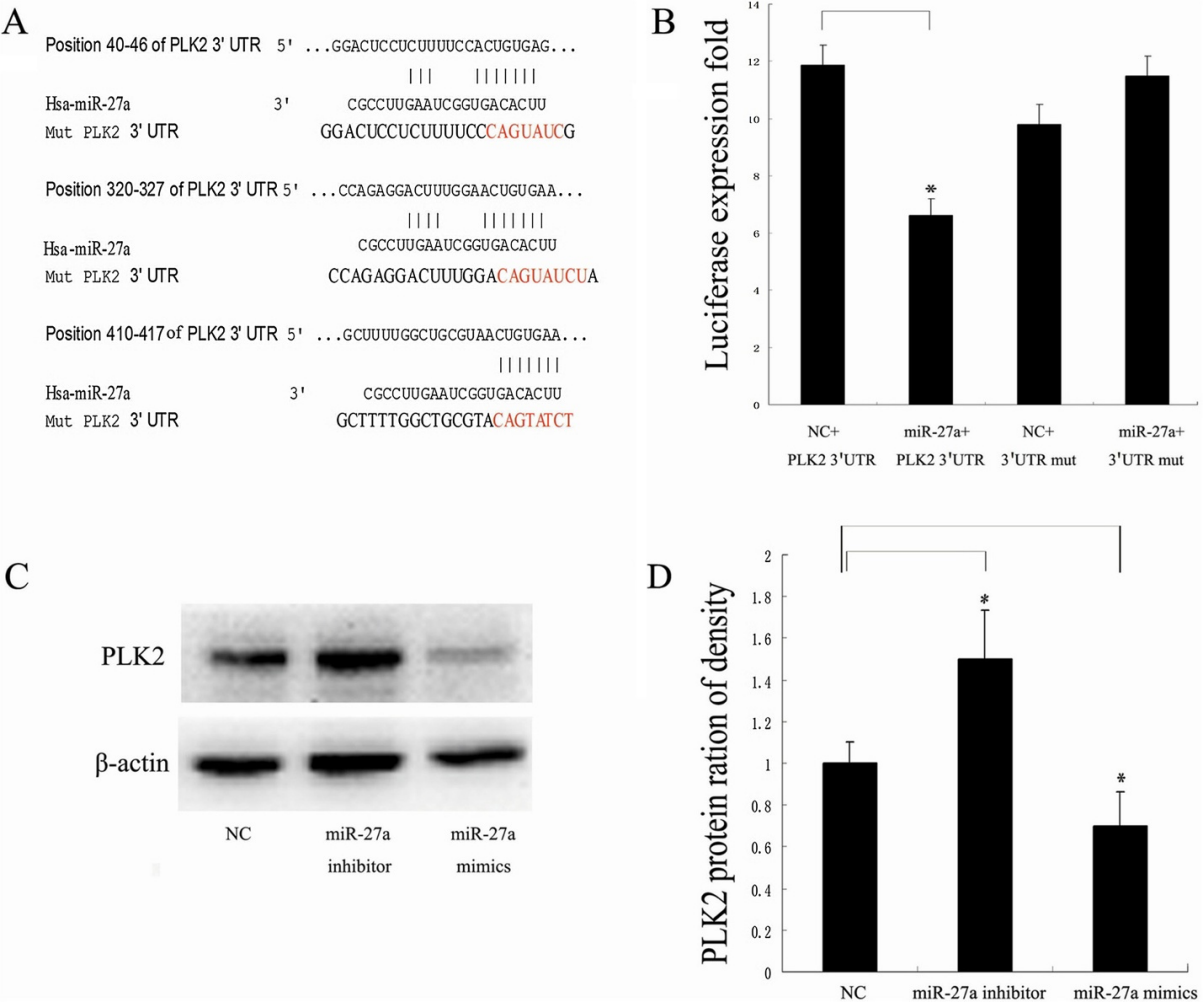
**Validation of** ***PLK2*** **as a direct target of miR-27a. (A)** Putative miR-27a binding sites on 3’-UTR of *PLK2* mRNA. Three miR-27a binding sites on 3′-UTR of *PLK2* mRNA were predicted by the corresponding programs. The designed mutant nucleotides are highlighted in red color. **(B)** The luciferase activity in the HEK293 cells. HEK293 cells were cotransfected with different miRNAs and the luciferase activities were detected in different groups. Each value is evaluated by the relative luciferase activity of firefly to renilla. **(C)** Effect of miR-27a on *PLK2* protein level in the Hep2 cells. After the Hep2 cells were transfected, the *PLK2* protein expression was detected by Western blot. β-actin was used for the internal control. **(D)** Statistical analysis of the *PLK2* protein expression in the Hep2 cells. Data are the mean ± SD of three independent experiments. P < 0.05 is indicated as symbol*.

**Figure 4:**
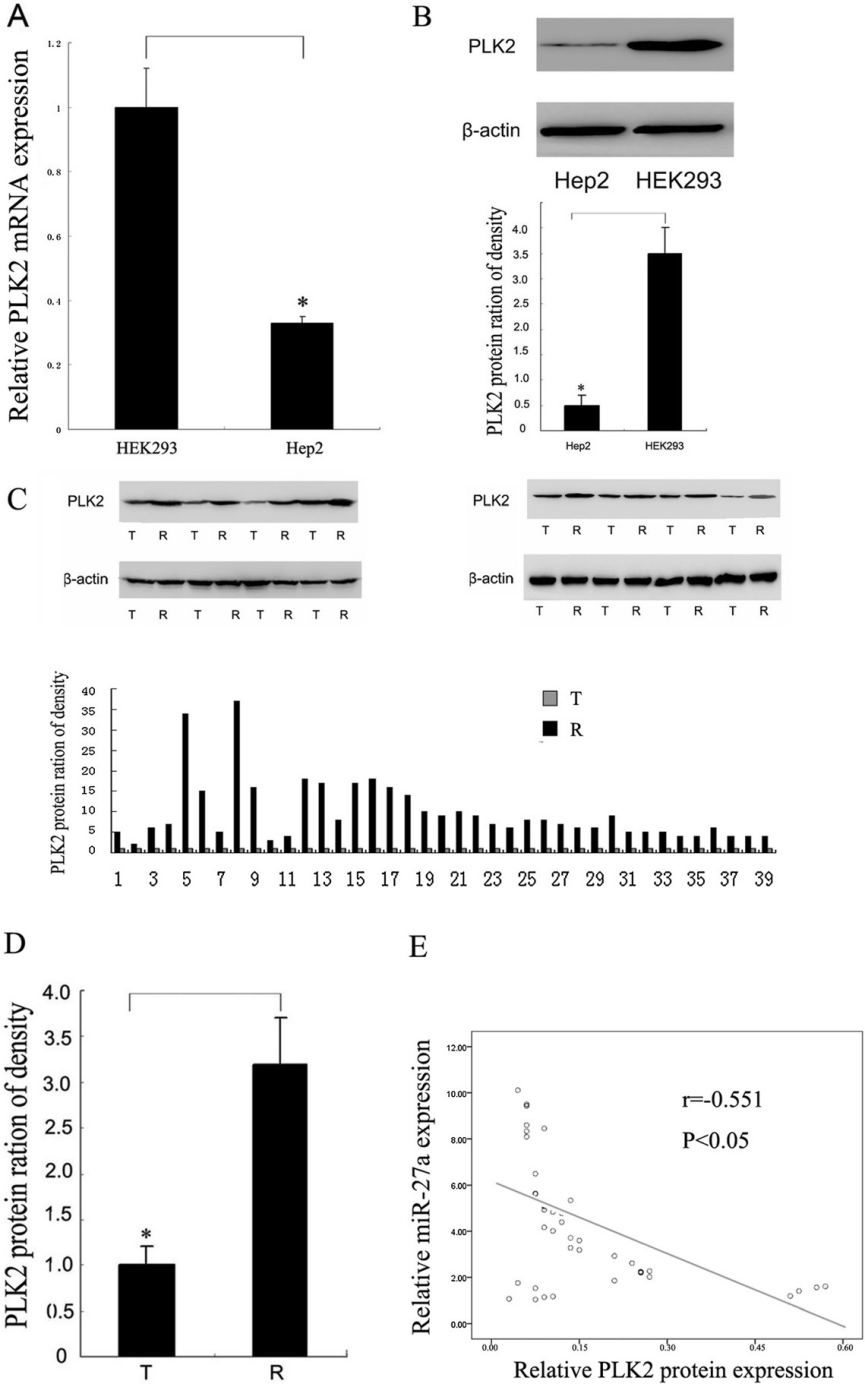
**Correlation between miR-27a and** ***PLK2*** **expression in LSCC. (A)** Relative expression of *PLK2* mRNA levels in the cell lines by qRT-PCR. The relative expression was calculated as the ratio of *PLK2* mRNA level to the internal control using the equation RQ = 2^–ΔΔCT^. **(B)** Relative expression of *PLK2* protein levels in the cell lines by Western blot. The relative expression level in each group is indicated as the ratio of *PLK2* to β-actin protein levels. **(C)** *PLK2* protein levels in LSCC tissues by Western blot. Up: Representative images of Western blot result. T and R indicate cancer tissue and the paired adjacent tissue, respectively. Down: Statistical analysis of relative expression of *PLK2* protein in each sample. The relative expression was calculated as the ratio of *PLK2* protein level in cancer tissue to that in the paired adjacent tissue in each case. The digit on X-axis show the number of the paired samples. **(D)** Statistical analysis of the *PLK2* protein expression in LSCC tissues. T and R indicate cancer tissue and the paired adjacent tissue, respectively. **(E)** Correlation between miR-27a and *PLK2* expression in LSCC by Pearson’s product–moment correlation coefficient. Data were expressed as the mean ± SD from three independent experiments. P < 0.05 is indicated as symbol*.

Based on the bioinformatics analysis using three different programs (miRanda, Pictar and targetscan), a highly-conserved miR-27a targeting sequence was predicted in the 3′-untranslated region of the *PLK2* mRNA (Figure 3A; see Additional file 3: Figure S3), which suggests that *PLK2* mRNA is a potential target of miR-27a. To confirm whether miR-27a directly targets the region of *PLK2*, dual luciferase reporter assays were carried out using the pGL3 construct in which *PLK2* 3′UTR fragment containing wild-type or mutant miR-27a binding sequence was cloned downstream of the firefly luciferase reporter gene (Figure 3A). As illustrated in Figure 3B, a significantly down-regulation on luciferase activity was found in the presence of miR-27a in the HEK293 cells cotranfected with pGL3-3′UTR of *PLK2*, but not with pGL3-3′UTR-mut. Western blot and real-time RT-PCR results indicated that miR-27a and its inhibitor significantly decreased and increased the *PLK2* expression at protein level (Figure 3C and D), but not at mRNA level (see Additional file 4: Figure S4), respectively. Both *PLK2* mRNA and protein levels were significantly lower in the Hep2 cells than those in the HEK293 cells (Figure 4A and B). In order to evaluate the relationship between miR-27a and *PLK2* expression levels, we detected *PLK2* protein level in 46 cases with miR-27a up-regulation by Western blot. As a result, 39 cases (70%) showed significant down-regulation of *PLK2* in cancer tissues compared to the controls (Figure 4C and D). The statistical analysis result revealed that miR-27a level was negatively correlated with PLK2 protein level in laryngeal cancer tissues (r = -0.551 P < 0.05; Figure 4E). These results suggest that miR-27a negatively regulates *PLK2* expression through the translational repression pathway.

### *PLK2*knockdown could induce the proliferation and inhibit the late apoptosis in Hep2 cells

**Figure 5:**
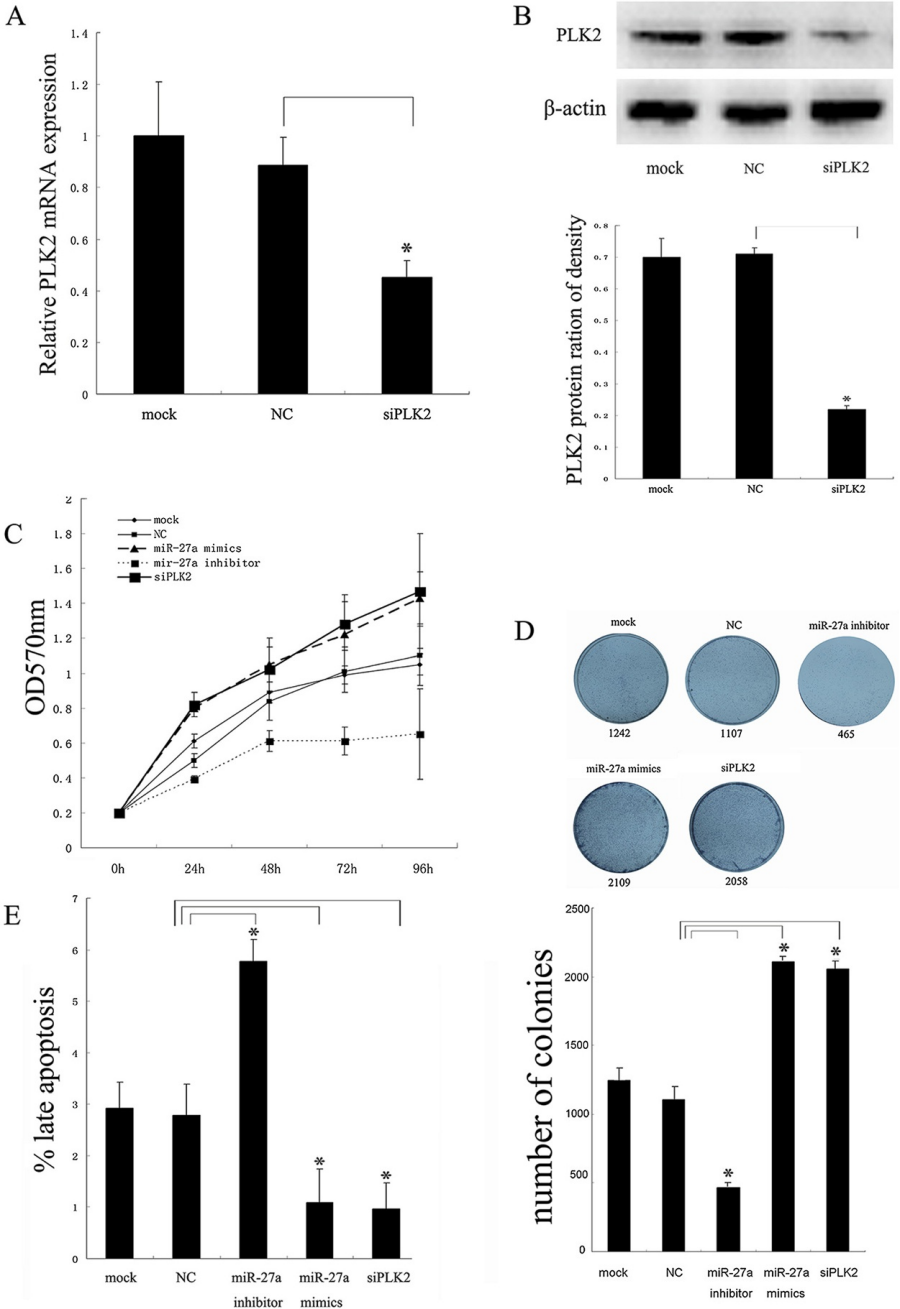
**Regulation of** ***PLK2*** **in the proliferation and apoptosis in the Hep2 cells. (A)** Relative *PLK2* mRNA levels in the Hep2 cells. Hep2 cells were silenced by siRNA-PLK2 and the *PLK2* mRNA was assayed by qRT-PCR. *GAPDH* was used as internal control. **(B)** Relative *PLK2* protein levels in the Hep2 cells. Hep2 cells were silenced by siRNA-PLK2 and the *PLK2* protein was detected by Western blot. β-actin was used as internal control. **(C)** Effect of siRNA-PLK2 on the Hep2 cell proliferation measured by the MTT assay. Hep2 cells were transfected with siRNA-PLK2 or the control miRNAs in the Hep2 cells and the cell proliferation was detected using the MTT assay. **(D)** Effect of siRNA-PLK2 on the Hep2 cell proliferation measured by the colony formation assay. Hep2 cells were transfected with siRNA-PLK2 or the control miRNAs in the Hep2 cells and the cell proliferation was detected using the colony formation assay. **(E)** Effect of siRNA-PLK2 on the late apoptosis of the Hep2 cell lines. Hep2 cells were transfected with siRNA-PLK2 or the control miRNAs and treated by Annexin V-EGFP apoptosis detection kit. The late apoptotic percentages of the Hep2 cells in different groups were monitored by flow cytometry. Data were expressed as the mean ± SD from three independent experiments. P < 0.05 is indicated as symbol*.

To evaluate whether inhibition of *PLK2* plays the similar role in the regulation of the proliferation and apoptosis as miR-27a does in the Hep2 cells, *PLK2* were silenced by its siRNA and the effect on the proliferation and apoptosis was detected. As shown in Figure 5A and B, the *PLK2* gene was significantly inhibited by its siRNA both at mRNA and protein levels in the Hep2 cells, respectively. The MTT and colony formation assay results showed that knockdown of *PLK2* significantly promoted cell viability and colony formation compared to the control groups (Figure 5C and D). Flow cytometry assay results indicated that knockdown of *PLK2* could significantly suppress the late apoptosis compared to the control groups (Figure 5F), but not the early apoptosis (see Additional file 5: Figure S5).

## Discussion

miR-27a is a family member of miR-23a ~ 27a ~ 24-2 cluster. The oncogenic or suppressive role of the cluster decides its function in diseases. As for the expression levels of the three family members within the cluster in the pathological conditions, there exist different conclusions. In some diseases, for examples in acute lymphoblastic leukemia (ALL), acute myeloid leukemia (AML) and cardiac hypertrophy, all the three members are highly expressed. In acute promyelocytic leukemia (APL), the three members are down-regulated. In prostate cancer, up-regulation of the members are found in one study and down-regulation in another one [32].

In the study, 67 paired of laryngeal cancer tissues were used. We found that miR-27a is significantly over-expressed in general in LSCC even though down-regulated in some cancer tissues. In our group, we also detected the expression of miR-23a and miR-24-2 in LSCC. The results showed that the two members are significantly up-regulated in general in LSCC (data not shown). These results suggest that miR-27a together with other two members play an important role as a potential oncogene in LSCC.

Identification of miR-27a target genes is important for our better understanding the role of miR-27a in tumorigenesis. At presence, some genes such as *ZBTB10/RINZF* [25], *FOXO1* [26], and *FBW7* [33] have been confirmed as targets of the miR-27a gene. Based on the bioinformatics searching results, miR-27a has 1211 conserved targets (data not shown). According to the expression level of mir-27a in LSCC, we have selected a presumable tumor suppressor gene *PLK2* as a potential target of miR-27a among the predicted genes. Until now, miR-126, the only microRNA has been verified to directly regulate the *PLK2* gene [34]. In the present study, we found that miR-27a represses the expression level of *PLK2* in the Hep2 cells. We also found that miR-27a inhibits the luciferase activity of the reporter in the HEK293 cells cotransfected with wild-type *PLK2*-3′UTR, but not that with the mutant *PLK2-*3′UTR. In addition, there exists the negative correlation between miR-27a and *PLK2* expression levels in laryngeal cancer tissues. These results demonstrate that *PLK2* is the direct target gene of miR-27a.

*PLK2* belongs to Polo-like kinase (*PLK*) family which includes *PLK1, PLK 2 (Snk), PLK 3 (Fnk, Prk)* and *PLK 4 (Sak)*. PLK proteins play critical roles in the control of cell cycle progression, either favoring or inhibiting cell proliferation, and in DNA damage response. In human hepatocellular carcinoma (HCC), PLK1 acts as an oncogene and PLK2-4 presumably tumor suppressor genes [35]. In some studies, however, PLK2 promotes the cell proliferation in the SKBR3 cells (breast cancer) and primary keratinocyte, respectively [36, 37], which suggests that the gene plays an oncogenic role. Our present results indicated that transfection of miR-27a and silence of *PLK2* by its siRNA could induce the proliferation and colony formation of the Hep2 cells, which suggests miR-27a targets *PLK2*, leading to the proliferation and colony formation in the Hep2 cells. In other researches, miR-27 promotes proliferation via different targets. For examples, miR-27a enhances myoblast proliferation by targeting myostatin [38]. miR-27a increases the growth and colony formation of pancreatic cancer cells by targeting Sprouty2 [39].

Syed *et al.* demonstrated that ectopic expression of *Snk*/ *PLK2* in BL cells results in apoptosis and loss of *Snk*/ *PLK2* expression was one of the most common events in B-cell neoplasia, strongly supporting that *PLK2* is a human tumor suppressor gene [40]. Burns et al. found that small interfering RNA (siRNA)-mediated *Snk*/*PLK2* silencing significantly increased apoptosis in the osteosarcoma, non-small cell lung cancer and cervical carcinoma cells [41]. Our present results indicated that miR-27a itself and silence of *PLK2* by its siRNA could cause the repression of the late apoptosis in the Hep2 cells, which suggests miR-27a targets *PLK2*, resulting in the late apoptosis in the Hep2 cells. In addition, miR-27a also inhibits the early apoptosis of the Hep2 cells in the study, which has not been reported yet. Therefore, this mechanism of the novel finding needs to be further investigated.

In our study, we also found that miR-27a does not affect the cell cycle of the Hep2 cells. However, miR-27a increases the percentage in G(2)-M in MDA-MB-231 breast cancer cells by targeting Myt-1 [25]. We speculate that the different cancer types contribute to the diverse response to miR-27a.

## Conclusions

We first reveal that miR-27a plays an oncogenic role in LSCC. This function in LSCC is associated, in part, with the inhibition of *PLK2*. miR-27a could be a potential target for the gene diagnosis and therapy of LSCC.

## Electronic supplementary material

Additional file 1: Figure S1: miR-27a expression in LSCC and Hep2 cells by qRT-PCR. (A) Amplification plot of miR-27a. (B) Dissociation curve of miR-27a. (JPEG 423 KB)

Additional file 2: Figure S2: Effect of miR-27a on the Hep2 cell cycle. Hep2 cells were transfected with miR-27a or the control miRNAs and the Hep2 cell cycle in different groups were monitored by flow cytometry. Data were expressed as the mean ± SD from three independent experiments. P < 0.05 is indicated as symbol*. (JPEG 160 KB)

Additional file 3: Figure S3: The alignment of the miR-27a targeting sequences located in the 3′-UTR of the *PLk2* genes from 23 organisms. The evolutionarily conserved nucleotides are indicated with capital letters in the sequence shown on the bottom. (JPEG 331 KB)

Additional file 4: Figure S4: Effect of miR-27a on *PLK2* mRNA level in the Hep2 cells. After the Hep2 cells were transfected, the *PLK2* mRNA expression was detected by qRT-PCR. The relative expression was calculated as the ratio of miR-27a to the internal control using the equation RQ = 2^–ΔΔCT^ in each sample. Data were expressed as the mean ± SD from three independent experiments. P < 0.05 is indicated as symbol*. (JPEG 171 KB)

Additional file 5: Figure S5: Effect of si-PLK2 on the early apoptosis of the Hep2 cells. Hep2 cells were transfected with si-PLK2 or the control miRNAs and treated by Annexin V-EGFP apoptosis detection kit. The early apoptotic percentages of the Hep2 cells in different groups were monitored by flow cytometry. Data were expressed as the mean ± SD from three independent experiments. P < 0.05 is indicated as symbol*. (JPEG 138 KB)
