# Supplementary figures and images for: MicroRNA-27a promotes proliferation and suppresses apoptosis by targeting PLK2 in laryngeal carcinoma
Source: BMC Cancer. 2014 Sep 18;14:678. doi: 10.1186/1471-2407-14-678 (PMC4177177; doi:10.1186/1471-2407-14-678)

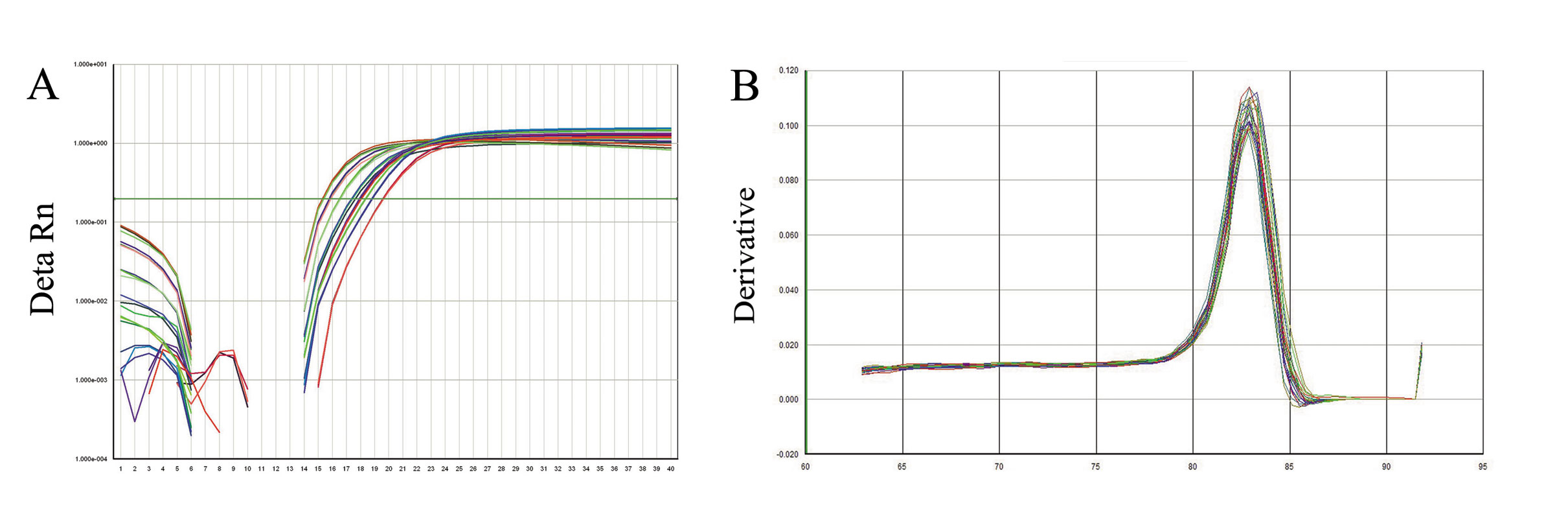

Supplement: Supplementary file 1 — Additional file 1: Figure S1: miR-27a expression in LSCC and Hep2 cells by qRT-PCR. (A) Amplification plot of miR-27a. (B) Dissociation curve of miR-27a. (JPEG 423 KB) [file 12885_2013_4857_MOESM1_ESM.jpeg]

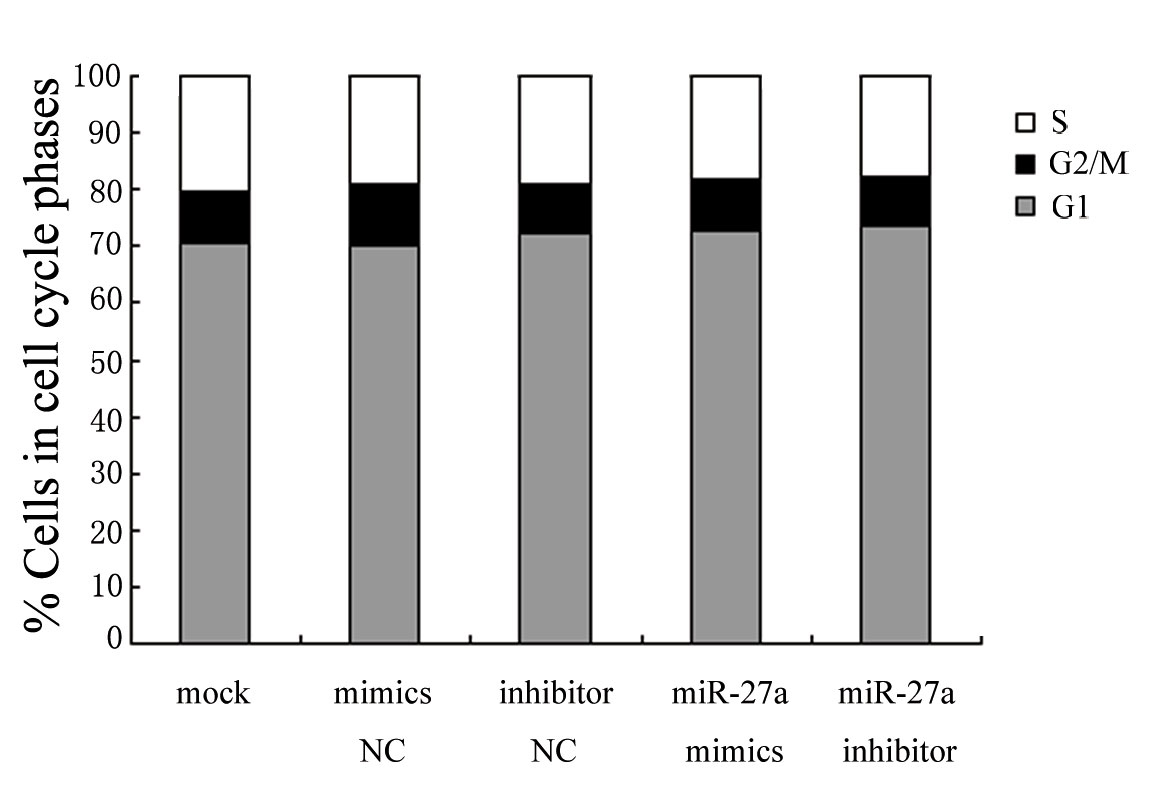

Supplement: Supplementary file 2 — Additional file 2: Figure S2: Effect of miR-27a on the Hep2 cell cycle. Hep2 cells were transfected with miR-27a or the control miRNAs and the Hep2 cell cycle in different groups were monitored by flow cytometry. Data were expressed as the mean ± SD from three independent experiments. P < 0.05 is indicated as symbol*. (JPEG 160 KB) [file 12885_2013_4857_MOESM2_ESM.jpeg]

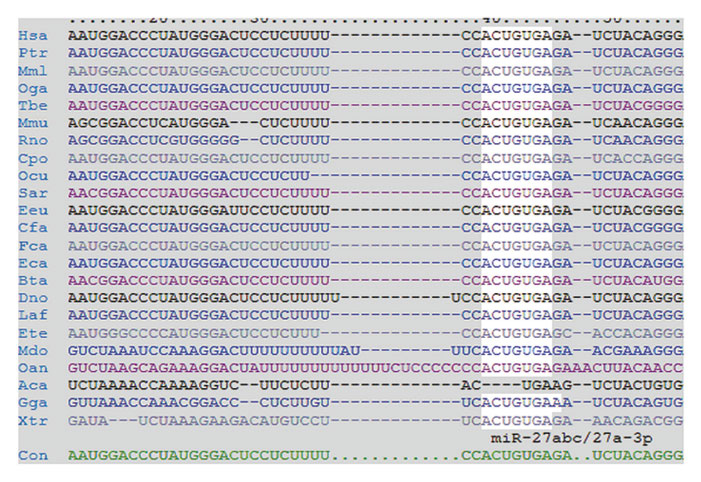

Supplement: Supplementary file 3 — Additional file 3: Figure S3: The alignment of the miR-27a targeting sequences located in the 3′-UTR of the PLk2 genes from 23 organisms. The evolutionarily conserved nucleotides are indicated with capital letters in the sequence shown on the bottom. (JPEG 331 KB) [file 12885_2013_4857_MOESM3_ESM.jpeg]

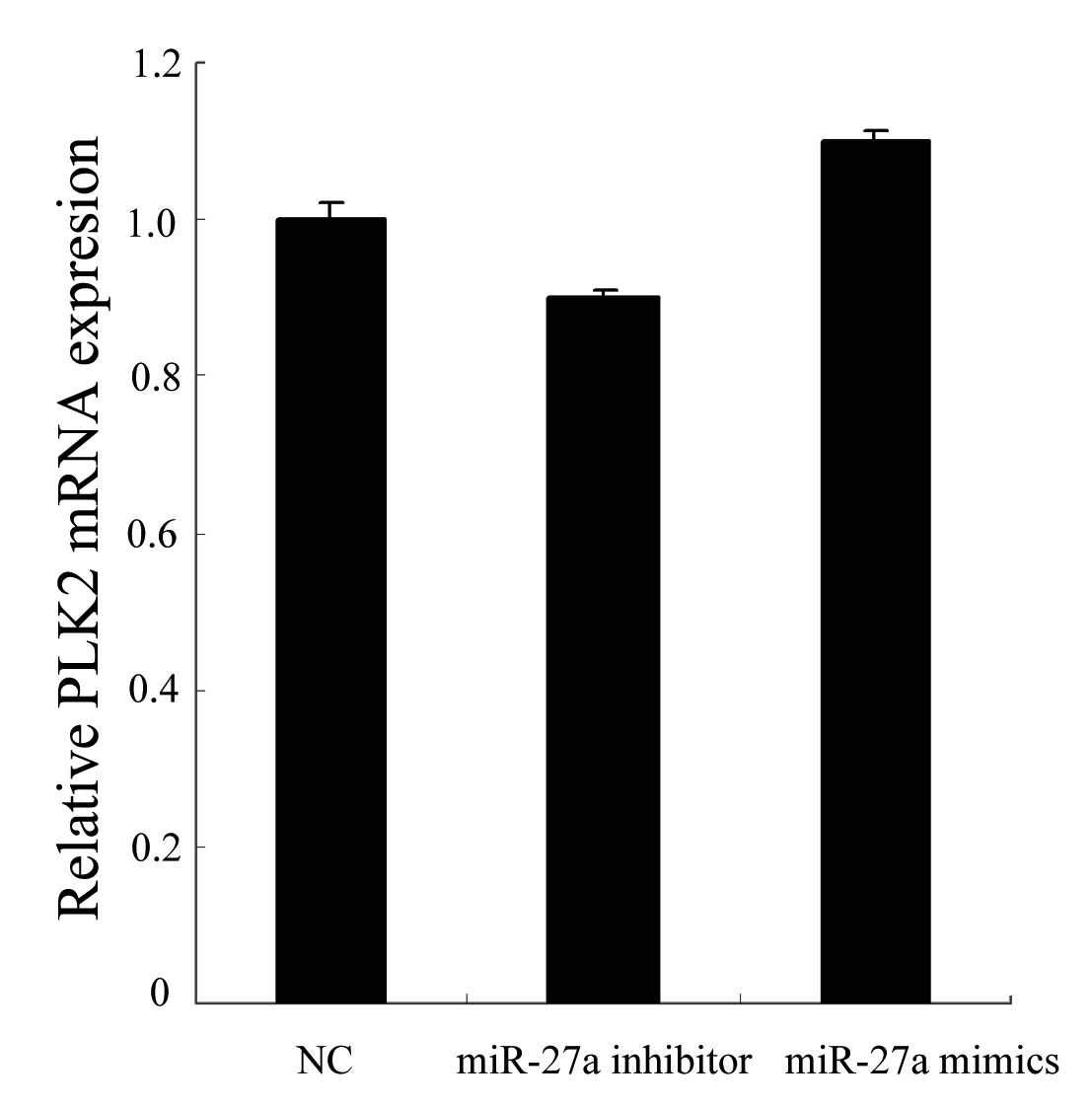

Supplement: Supplementary file 4 — Additional file 4: Figure S4: Effect of miR-27a on PLK2 mRNA level in the Hep2 cells. After the Hep2 cells were transfected, the PLK2 mRNA expression was detected by qRT-PCR. The relative expression was calculated as the ratio of miR-27a to the internal control using the equation RQ = 2–ΔΔCT in each sample. Data were expressed as the mean ± SD from three independent experiments. P < 0.05 is indicated as symbol*. (JPEG 171 KB) [file 12885_2013_4857_MOESM4_ESM.jpeg]

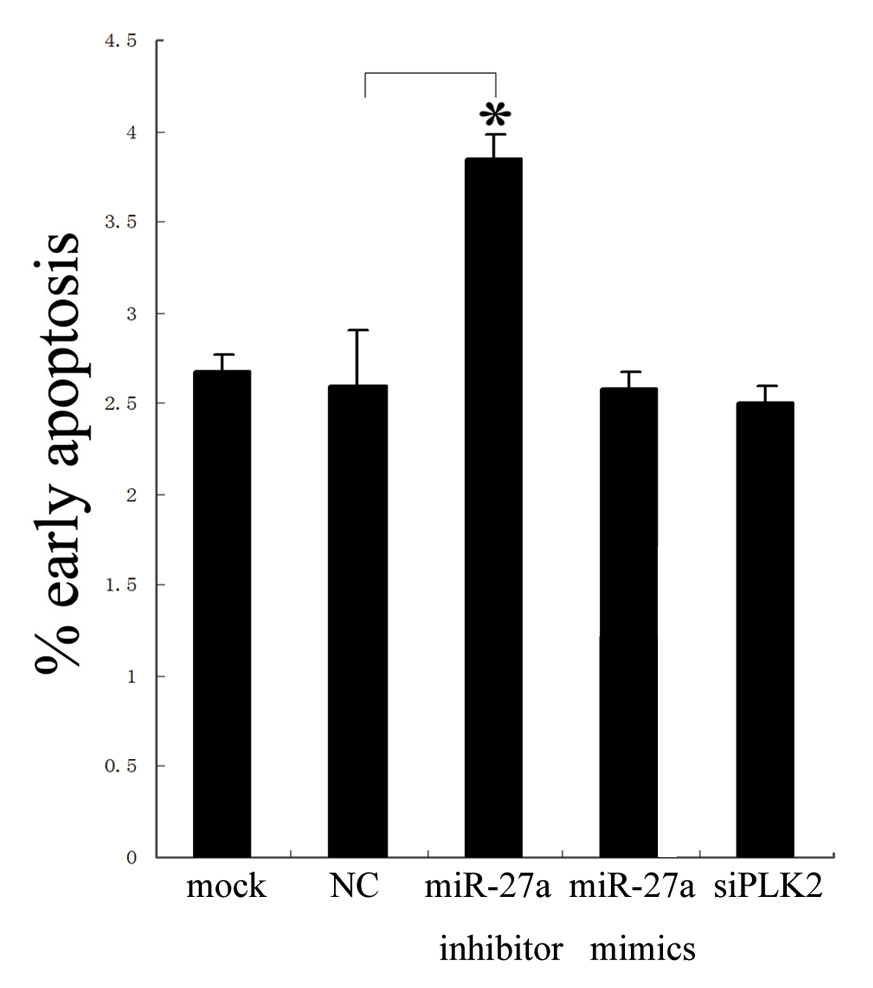

Supplement: Supplementary file 5 — Additional file 5: Figure S5: Effect of si-PLK2 on the early apoptosis of the Hep2 cells. Hep2 cells were transfected with si-PLK2 or the control miRNAs and treated by Annexin V-EGFP apoptosis detection kit. The early apoptotic percentages of the Hep2 cells in different groups were monitored by flow cytometry. Data were expressed as the mean ± SD from three independent experiments. P < 0.05 is indicated as symbol*. (JPEG 138 KB) [file 12885_2013_4857_MOESM5_ESM.jpeg]
